# The amplitude of pulse-synchronous oscillations varies with the level of intramuscular pressure in simulated compartment syndrome

**DOI:** 10.1186/s40634-015-0020-6

**Published:** 2015-02-25

**Authors:** Andreas Nilsson, Qiuxia Zhang, Jorma Styf

**Affiliations:** Department of Orthopaedics, Institute of Clinical Sciences, Sahlgrenska Academy, University of Gothenburg, Sahlgrenska University Hospital, Guldhedsgatan 10 A, 413 46 Gothenburg, Sweden; Department of Orthopaedics, Sahlgrenska University Hospital, Gothenburg, Sweden

**Keywords:** Intramuscular pressure, Pulse-synchronous oscillations in intracompartmental pressure, Compartment syndrome, Intramuscular arterial pulsations, Fiber-optic technique

## Abstract

**Background:**

Patients with compartment syndromes have elevated intramuscular pressure (IMP) due to increased volume in the affected muscle. However, the accuracy of IMP as a parameter in diagnosing chronic compartment syndrome has been questioned. It has been observed that arterial pulsations create oscillations in the IMP in patients with abnormally elevated IMP. The amplitude of the IMP oscillations appears to be related to a pathogenic mechanism of elevated IMP. Therefore, the purpose of the present study was to investigate the relation between the amplitude of pulse-synchronous IMP oscillations and the absolute level of IMP with a high-end fiber-optic system in a human experimental model of abnormally elevated IMP (simulated compartment syndrome) of the leg. The hypothesis that the amplitude of the IMP oscillations is correlated to the absolute level of IMP was tested.

**Methods:**

IMP was measured at rest in the anterior tibial muscle in 12 legs of 7 healthy subjects (4 females and 3 males) with a mean age of 28 (range 23–38) years. The subject lay supine with his/her heel placed in a footrest. The foot was kept in a neutral position to avoid biased IMP readings. Measurements were performed at baseline and during 10 minutes with a model of abnormally elevated IMP (simulated compartment syndrome) applied. The abnormally elevated IMP was created by venous obstruction induced by a thigh tourniquet (65 mmHg) of a casted leg. Placement of the pressure-recording catheter was verified by sonography.

**Results:**

The IMP increased from 4.7 (SD = 1.8) mmHg at baseline to 48.6 (SD = 7.1) mmHg when the model of elevated IMP was applied. The amplitude of the pulse-synchronous oscillations was undetectable at baseline. It increased to 3.9 (SD = 1.4) mmHg with increasing IMP when the model was applied. The amplitude of the oscillations showed a positive correlation (r = 0.59) with the absolute level of IMP.

**Conclusions:**

The amplitude of the pulse-synchronous IMP oscillations is correlated with the absolute level of IMP during abnormally elevated IMP. The oscillations of IMP may therefore be an additional parameter assuring the abnormally elevated IMP in the diagnosis of compartment syndromes.

## Background

Intramuscular pressure (IMP) is often regarded as the gold standard in the diagnosis of compartment syndromes (Matsen et al. 1976; Hargens et al. 1977; Hargens et al. 1989), since clinical findings without the support of IMP measurements have low sensitivity (Balduini et al. 1993). IMP measurements may be acquired at rest before, during, and at rest after exercise (Styf and Körner 1987). However, several IMP criteria have been suggested and the validity and the evidence for the commonly used pressure criteria in diagnosing chronic compartment syndrome have recently been under question (Tiidus 2014; Aweid et al. 2012; Franklyn-Miller et al. 2012).

Patients with compartment syndrome have an elevated IMP due to increased volume in the affected muscle (Eliassen et al. 1974; Styf and Körner 1987). The volume load reduces the compliance of the compartment. Reduced compliance implies that a small increase in volume in the muscle causes a large increase in IMP. Arterial pulsations create oscillations in the IMP in patients with decreased compliance and abnormally elevated IMP (Styf and Körner 1986). The amplitude of the IMP oscillations appears to be related to a pathogenic mechanism of elevated IMP and it varies with the arterial pulsations and the change in the compliance of the compartment (Styf 1995; Garabekyan et al. 2009; Lynch et al. 2009, Nilsson et al., 2014). The mean amplitude of the IMP oscillations has been reported to be approximately 5 mmHg in patients with chronic anterior compartment syndrome and less than 1 mmHg in patients without chronic anterior compartment syndrome at rest after an exercise test (Styf and Korner 1986). There are, however, no reports establishing the potential relationship between the amplitude of the oscillations and the absolute level of IMP. Therefore, the purpose of the present study was to investigate the amplitude of pulse-synchronous IMP oscillations relation to the absolute IMP with a high-end fiber-optic pressure measurement system in an experimental model of elevated IMP (simulated compartment syndrome) in the human leg. The hypothesis that the amplitude of the IMP oscillations is correlated to the absolute level of IMP was tested.

## Methods

### Subjects

Seven healthy subjects (four females and three males) with a mean age of 28 (range 23–38) years and a mean body mass index (BMI) of 23 (range 20–26) kg/m^2^ volunteered to participate in the study. The study was performed on 12 legs of these subjects. All the subjects gave their informed written consent prior to participation. The study protocol was approved by the regional Research Ethics Committee.

### IMP recordings

IMP was recorded for 5 minutes for baseline data, 10 minutes during a model of abnormally elevated IMP (simulated compartment syndrome), followed by 5 minutes of recovery after the model was removed. The subject lay supine with his/her heel placed in a padded footrest. The function of the footrest was to keep the leg at heart level and to ensure that no external compression from the bed was applied to the calf. The heart level was defined as 5 cm below the manubrium sterni. The foot was kept in a relaxed neutral position to avoid effects on the IMP (Weiner et al. 1994). Measurements were made in both legs in five subjects but on different occasions. In two subjects, measurements were only made in one leg.

### IMP measurement systems

A fiber-optic pressure transducer with a diameter of 0.42 mm and an estimated volume of 0.072 mm^3^ (Samba 420 LP, Samba Sensors AB, Göteborg, Sweden) was used for the IMP measurement. The sensing area is placed at the distal end of the fiber and is forward sensing. The transducer was connected to a Samba 3200 control unit (Samba Sensors AB, Göteborg, Sweden) set to measure relative pressure in mmHg. The transducer was calibrated at room temperature before insertion. The Samba control unit was connected to a computer (PC) equipped with data acquisition hardware from National Instruments (National Instruments, Austin, TX, USA) and custom-made LabView based software. All units were turned on at least 30 minutes before the start of IMP measurements to minimize internal temperature drift. Data were collected at 20 Hz. In addition, a Stryker Intra-Compartmental Pressure Monitor was used with a 1.3 × 60 mm disposable needle with a sideport and interconnected with tubing. The 20 cm long tubing and a 3 ml syringe were prefilled with room-tempered saline. During measurements, the transducer of the monitor was kept at the same level as the tip of the needle to avoid hydrostatic offset. The IMP values from the Stryker system were used as a reference and were not analyzed further.

### Model of abnormally elevated IMP (simulated compartment syndrome)

The IMP was abnormally elevated by venous obstruction induced by a thigh tourniquet on a casted leg. The plaster cast was applied over two layers of cotton padding extending from slightly below the knee joint to the distal part of the leg. The proximal part of the plaster was modified to make sufficient room for two catheters used for IMP measurements. A 145 mm wide pneumatic tourniquet was placed around the thigh and inflated to 65 mmHg to obstruct venous return from the leg.

This method of elevating IMP has previously been evaluated in healthy subjects (Styf and Wiger 1998) and used to simulate compartment syndromes in the human leg (Zhang et al. 2001; Wiger and Styf 1998; Wiger et al. 2000; Styf and Wiger 1998). The model elevated IMP to levels that are seen in patients with compartment syndromes and elicited sensory dysfunction, muscle weakness, a mild throbbing pain and oscillations of the IMP (Styf and Wiger 1998). In this study, the model was applied for only 10 minutes to elevate the IMP without provoking any additional symptoms associated with compartment syndromes.

### Measurement catheter insertion

A local anesthetic (1–2 ml of xylocain 10 mg/ml with epinephrine, 5 μg/ml) was injected subcutaneously approximately 7 cm below the knee joint and 2 cm lateral to the tibial tuberosity of the test leg. Under sterile conditions, a Venflon introducer (1.3 × 45 mm) was inserted through the skin into the anterior tibial muscle fascia in a distal direction at an angle of 30 degrees from the plane of the skin, while the subject kept his/her ankle joint dorsiflexed. The tip of the needle was then retracted into the sheath of the introducer and the set was bluntly advanced parallel to the muscle fibers in the relaxed muscle with the foot in a neutral position. The angle of insertion was kept as parallel to the muscle fibers as possible to reduce pain, trauma to the muscle and local edema that may affect pressure measurements (Styf 1989). The needle was then removed and the optic fiber was inserted 45 mm into the Venflon tubing. The sensor element of the optic fiber was thereby positioned at the end of the tubing. A Luer lock Tuohy Borst Adapter with a gasket ensured sealing and fixation of the optic fiber. The Stryker needle was inserted using the same procedure but 10 mm lateral or medial (every other time) and parallel to the optic fiber. Since IMP varies with depth (Nakhostine, 1993), both transducers were introduced to the same depth. The depth of the catheter tip and the angle between the catheter and the overlying fascia were measured by sonography (Acuson CV-70, Siemens Medical Solutions USA, Inc., CA, USA).

### Blood pressure and pulse rate

Blood pressure and pulse rate were measured repeatedly with a non-invasive blood pressure manometer (NAIS, Matsushita, Electronic Works, Japan) in the left arm. Mean arterial pressure (MAP) was calculated by adding one third of the pulse pressure to the diastolic blood pressure. Local perfusion pressure was calculated as MAP minus IMP.

### Statistical method

Wilcoxon signed rank test was used for comparisons and significance was set at p < 0.05. Correlations are given with Pearson’s r. Unless otherwise stated, all results are given as the mean and standard deviation (SD).

## Results

**Figure 1:**
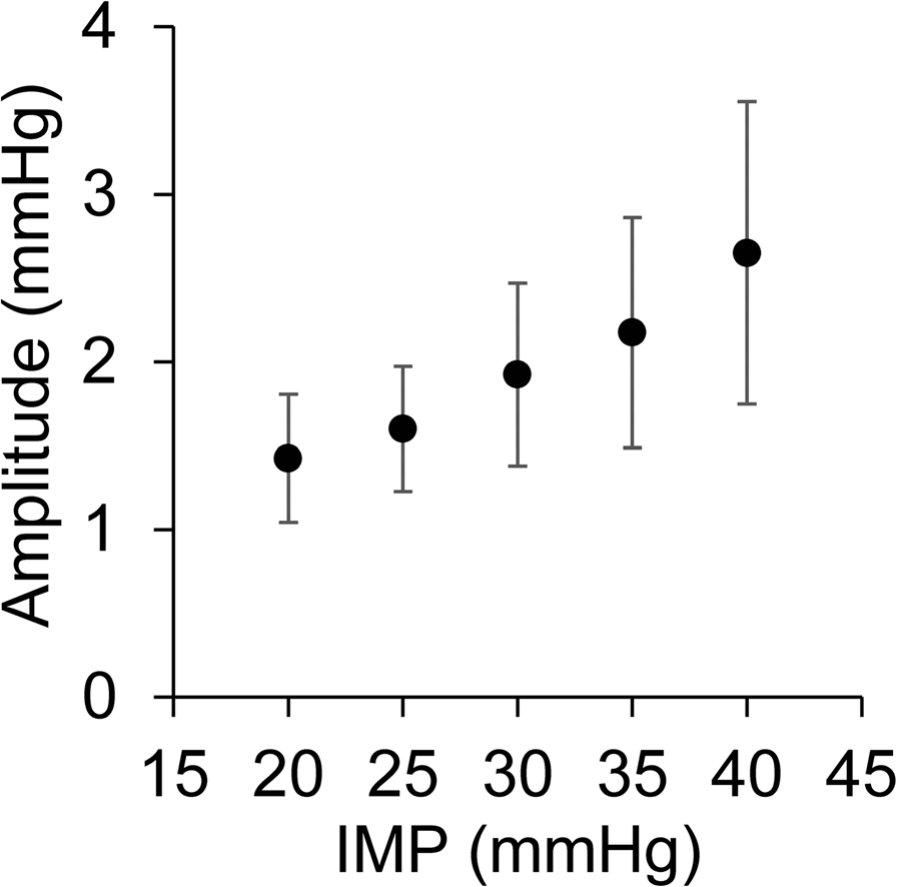
**The amplitude of the pulse-synchronous oscillations.** The mean amplitude of the pulse-synchronous oscillations increased with the absolute level of the intramuscular pressure (IMP) when the model of abnormally elevated IMP (simulated compartment syndrome) was applied. Error bars = standard deviation.

**Figure 2:**
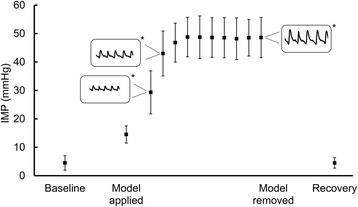
**Intramuscular pressure during a model of simulated compartment syndrome.** Mean intramuscular pressure (IMP) and standard deviation during a model of abnormally elevated IMP that was induced by venous obstruction of a casted leg. Oscillations in the IMP are schematically visualized (*). The peak-to-peak amplitude of the oscillations increased as a function of the IMP.

The IMP at baseline was 4.7 (SD = 1.8) mmHg and no oscillations that were synchronous with the arterial pulse could be detected. When the plaster cast was applied, the IMP increased to 15 mmHg and when the tourniquet was inflated to 65 mmHg, the IMP increased to over 40 mmHg within two minutes. The peak-to-peak amplitude of the IMP oscillations was analyzed for IMP values between 20 and 40 mmHg (Figure 1). The amplitude of the oscillations showed a positive correlation (r = 0.59, p < 0.01) to the level of the IMP. After 10 minutes with the model of abnormally elevated IMP (simulated compartment syndrome) applied, the IMP was 48.6 (SD = 7.1) mmHg and the amplitude of the IMP oscillations was 3.9 (SD = 1.4) mmHg (Figure 2). During recovery, the IMP returned to baseline and the amplitude was no longer detectable.

The local perfusion pressure decreased to 33 mmHg (44% of the baseline value) during the model of abnormally elevated IMP (p < 0.01), while the mean arterial pressure and pulse rate were essentially unchanged throughout the experiment (p > 0.05) (Table 1).

**Table 1:** Mean arterial pressure, local perfusion pressure and pulse rate

	**MAP**		**PP**		**Pulse rate**	
	**mmHg**	**SD**	**mmHg**	**SD**	**bpm**	**SD**
Baseline	80	5	75	5.1	62	7
Elevated IMP	82	5	33	9.5	61	9
Recovery	82	7	78	7.6	59	7

Mean arterial pressure (MAP), local perfusion pressure (PP) calculated from the intramuscular pressure (IMP) and pulse rate measured at baseline, during a model of abnormally elevated IMP (simulated compartment syndrome) and recovery.

The distance between the muscle fascia and the tip of the fiber-optic catheter was 6.6 (SD = 1.7) mm and the pennation angle was 9.4 (SD = 2.4) degrees.

## Discussion

Our study shows that the amplitude of the pulse-synchronous IMP oscillations is associated with the absolute level of IMP during abnormally elevated IMP. No pulse-synchronous IMP oscillations were detected at baseline or during recovery (normal IMP).

The amplitude of the IMP oscillations has been reported to decrease significantly from 4.9 (SD = 2.7) mmHg before surgery to 1.0 (SD = 0.6) mmHg following fasciotomy in patients with chronic anterior compartment syndrome (Styf and Korner 1986). The amplitude has been reported to be 5.8 (SD = 2.7) in patients with chronic anterior compartment syndrome and less than 1 mmHg or not detectable in patients without chronic anterior compartment syndrome (Styf and Körner 1987). The recorded amplitudes in the present study are in line with the above-mentioned studies on abnormally elevated IMP. The positive correlation between the amplitude and the absolute IMP level found in the current study indicates that the amplitude may be used to verify the diagnosis of compartment syndromes.

The increased IMP during simulated compartment syndrome in the present study reduced the muscle compliance and arterial pulsations were then reflected by pulse-synchronous oscillations in the IMP. The shape of these oscillations was similar to the typical pulsations that are seen in clinical invasive measurements of intra-arterial pressure. The waveform of IMP oscillations has been investigated with non-invasive ultrasound (Lynch et al. 2009). A preliminary report showed that frequency analysis of the fascial displacement waveform revealed a linear correlation between the ratio of the amplitude of the fundamental frequency and the second harmonic frequency and the invasively measured IMP (Wiemann et al. 2006). On the other hand, in an experimental porcine model of acute compartment syndrome, the analysis of the waveform revealed no significant interaction (Garabekyan et al. 2009). Since the amplitude was correlated to the IMP in the present study, the proposal that the proportion of the arterial pressure pulse that transmits through the muscle and affects the displacement of the compartment fascia is a function of the IMP (Garabekyan et al. 2009) is supported. Our results also support the concept that the oscillations might be useful for indirect measurements of the IMP using non-invasive techniques, such as ultrasound (Lynch et al. 2009).

It has been suggested that the thickness and elasticity of the fascia plays a role in the reduction in compartment compliance and the increase in IMP in patients with abnormally elevated IMP (Turnipseed et al. 1995; Hurschler et al. 1994; Detmer et al. 1985; Turnipseed et al. 1989). On the other hand, the stiffness and thickness did not differ between chronic compartment syndrome patients and healthy subjects in a recent study (Dahl 2011). Regardless of how the properties of the fascia influence compliance, the oscillations in the IMP may induce fascial displacement oscillations.

Local perfusion pressure around and below 30 mmHg has been reported to induce transient muscle ischemia (Heppenstall et al. 1988). During simulated compartment syndrome in our study, the local perfusion pressure decreased to 33 mmHg from 75 mmHg at baseline, while the mean arterial pressure and pulse rate were unchanged. Garabekyan and co-authors (Garabekyan et al. 2009) showed that fascial displacement was significantly larger in compartments with decreased perfusion pressure. These findings indicate that the oscillations increase with decreased perfusion pressure, since the perfusion pressure is a function of the IMP.

The experimental model of abnormally elevated IMP (simulated compartment syndrome) was chosen because it elevates the IMP by increasing the volume in the muscle compartment rather than by external compression. The IMP increased to approximately 50 mmHg, similar to previously reported values using this model (Styf and Wiger 1998; Wiger and Styf 1998; Zhang et al. 2001). This magnitude of IMP is also seen in patients with acute compartment syndrome (Taylor et al. 2012) and at rest after exercise in patients with chronic compartment syndrome (Aweid et al. 2012; Roberts and Franklyn‐Miller 2012). By applying the model for only 10 minutes the IMP was abnormally elevated but no other symptoms of compartment syndrome were provoked.

One limitation of this study is that the amplitude of the oscillations was not studied when the local perfusion pressure approached zero, as the IMP was only elevated to approximately 50 mmHg. One strength of this study is that the IMP was measured with a pressure recording system with excellent dynamic properties in a validated model of simulated compartment syndrome in the human leg. Nevertheless, our results need to be confirmed in patients with compartment syndromes.

## Conclusions

The amplitude of the pulse-synchronous IMP oscillations increases with increasing absolute level of IMP during a model of abnormally elevated IMP in the human leg. Since the amplitude is correlated with the absolute level of IMP, it may be an additional parameter in both research and in diagnosing compartment syndromes.
